# The impact of *COL1A1* and COL6A1 expression on hypospadias and penile curvature severity

**DOI:** 10.1186/s12894-020-00760-w

**Published:** 2020-12-01

**Authors:** Prahara Yuri, Gunadi, Rahmadani Puji Lestari, Firly Putri Fardilla, Wiwit Ananda Wahyu Setyaningsih, Nur Arfian, Ishandono Dachlan

**Affiliations:** 1 Division of Urology, Department of Surgery, Faculty of Medicine, Public Health and Nursing, Universitas Gadjah Mada/Dr. Sardjito Hospital, Jl. Kesehatan No.1, Yogyakarta, 55281 Indonesia; 2 Pediatric Surgery Division, Department of Surgery, Faculty of Medicine, Public Health and Nursing, Universitas Gadjah Mada/Dr. Sardjito Hospital, Yogyakarta, Indonesia; 3 Department of Anatomy, Faculty of Medicine, Public Health and Nursing, Universitas Gadjah Mada, Yogyakarta, Indonesia; 4 Division of Plastic, Aesthetic and Reconstructive Surgery, Department of Surgery, Faculty of Medicine,Public Health and Nursing, Universitas Gadjah Mada/Dr. Sardjito Hospital, Yogyakarta, Indonesia

**Keywords:** Chordee, *COL1A1*, *COL6A1*, Expressions, Hypospadias, Penile curvature severity, qPCR

## Abstract

**Background:**

Hypospadias, the most frequent congenital male external genitalia abnormality, is usually associated with curvature of the ventral penis, i.e. chordee. Abnormality of darto tissue has been suggested as the pathophysiology of chordees. Collagen is one of the most abundant fibrous proteins within the extracellular matrix. In this study, we determined the expression of *collagen 1 (COL1A1)* and *COL6A1* in patients with hypospadias and associated them with the severity of penile curvature.

**Methods:**

We included 60 children < 18 years old, consisting of 20 distal hypospadias, 20 proximal hypospadias patients, and 20 controls in our institution from 2017 – 2020. The expression of *COL1A1* and *COL6A1* in darto tissue was determined by reverse-transcriptase polymerase chain reaction (qPCR). The penile curvature severity was classified as mild (< 30 degrees), moderate (30–60 degrees), and severe (> 60 degrees).

**Results:**

qPCR showed that *COL1A1* and *COL6A1* expression was significantly downregulated in the distal (0.88 (0.38–2.53) and 0.54 (0.16–4.35), respectively) and proximal 0.76 (0.33–2.57) and 0.57 (0.18–1.38), respectively) hypospadias groups compared to controls (1.85 (0.24–4.61) and 0.93 (0.17–4.06), respectively) with *p*-values of 0.024 and 0.018, respectively. Furthermore, there was a moderate correlation between *COL1A1* and *COL6A1* expression (*r* = 0.458, *p* < 0.0001). Interestingly, *COL1A1* and *COL6A1* were also significantly downregulated in the moderate and severe chordee groups compared to the mild chordee groups, with *p*-values of 0.003 and 0.037, respectively.

**Conclusions:**

Aberrant *COL1A1* and *COL6A1* expression might affect abnormalities in darto tissue and penile curvature severity in hypospadias patients.

## Background

Hypospadias is the most frequent congenital penile anomaly that affects male external genitalia, with an incidence of approximately 1 in 250 male newborns. It is caused by tissue underdevelopment on the ventral aspect of the penis, resulting in an abnormal location of the urethral opening on the penile underside [1]. Elastosonography found deep alteration of hypospadias penile anatomy, in which the corpus spongiosum is stiffer and less elastic with less developed cavernous corpora [2].

Hypospadias is associated with curvature of the ventral penis, called the chordee. It is caused by the insufficiency or disorganization of the complex growth (the vascular and fascial structures) of the ventral penis [3]. Resection of dartos tissue usually straightens the penis in patients with chordee and buried penis, suggesting that the pathophysiology of these anomalies is related to dartos tissue [4].

The extracellular matrix (ECM) is a noncellular macromolecular network that supports cellular processes, such as proliferation, migration, differentiation, and homeostasis [5]. One of the most abundant fibrous proteins within the ECM is collagen. It has been considered the main component of the fibrous skeleton of the corpus cavernosum penis and is ubiquitous within the erectile tissues of the human penis [6, 7].

Fibrillar collagens are widely distributed in tissues providing tensile strength. Most fibrillar collagen is type 1 collagen, which can be found throughout the tissue beneath the urethral plate [6, 7]. Type 6 collagen (COL6A1) binds to type 1 collagen (COL1A1) to form thicker collagen fibers. COL6A1 is a part of the beaded filament-forming collagens and the most studied member of its group [8]. The dartos fascia in hypospadias is an abnormal tissue, characterized by inelastic and thick tissue, but it is not the same with fibrotic tissue [9]. Therefore, we determined the expression of *collagen 1 (COL1A1)* and *COL6A1* in patients with hypospadias and associated them with the severity of penile curvature.

## Methods

We included 60 children < 18 years old, consisting of 20 distal hypospadias, 20 proximal hypospadias patients, and 20 controls in our institution from 2017 – 2020. Detailed history and thorough examination of patients with hypospadias were performed by one urologist. We harvested periurethral darto tissue during urethroplasty in hypospadias patients and circumcision in normal penis samples.

### Reverse-transcriptase polymerase chain reaction (qPCR) assays

Fragments of the darto tissue were kept in RNAlater (Ambion, AM7021) solution before extraction. The tissue was extracted using Genezol RNA solution (GENEzol™, Cat. No. GZR100). cDNA was synthesized using an Excel RT Reverse Transcriptase Kit (RP1300, SMOBIO, Hsinchu City, Taiwan) with PCR conditions of 25 °C for 10 min (denaturation), 42 °C for 50 min (annealing), and 85 °C for 5 min (extension).

**Fig. 1:**
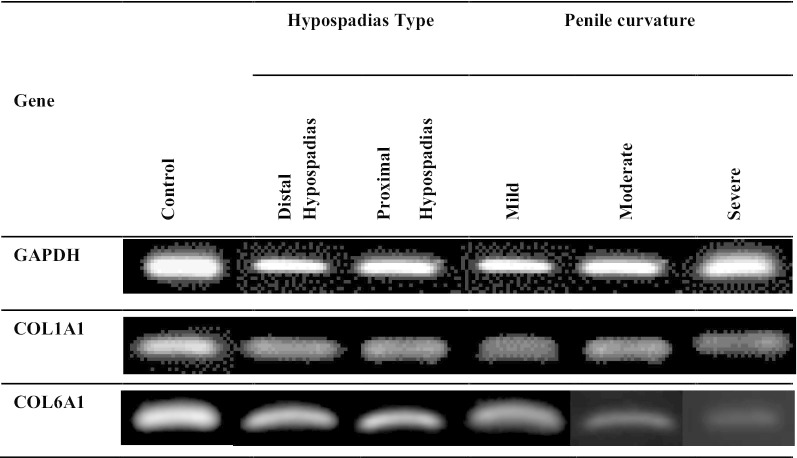
Representative of qPCR products of *COL1A1* and *COL6A1. GAPDH* was used as reference gene

**Fig. 2:**
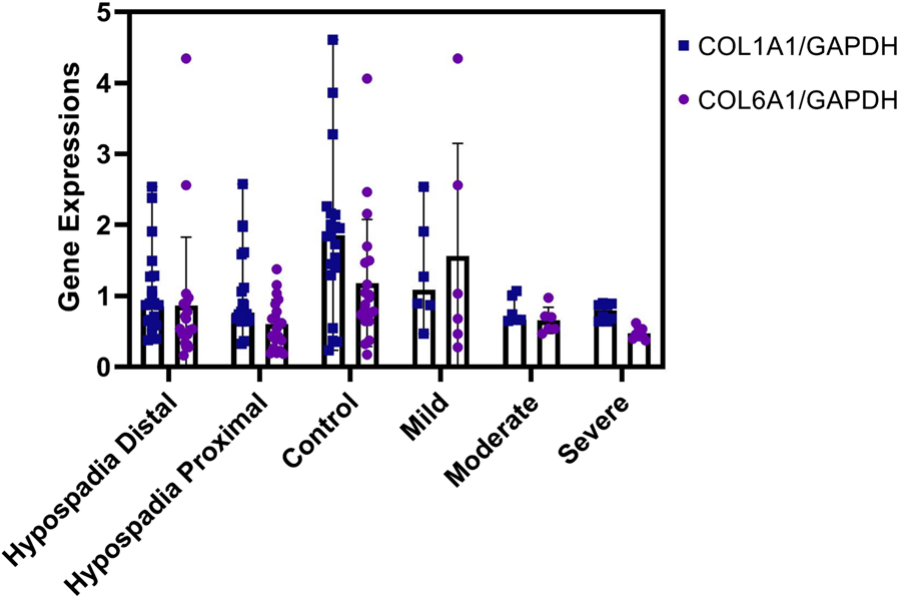
The *COL1A1* and *COL6A1* gene expressions in hypospadias patients and controls and among hypospadias groups, moderate and severe penile curvature

qPCR was performed by mixing the cDNA and Taq Master Mix (GoTaq®Green Master Mix, Cat. M7122) with the following primer genes: *COL1A1* (F: 5′-TACAGCGTCACTGTCGATGGC-3′ and R: 5′-TCAATCACTGTCTTGCCCCAG-3′), *COL6A1* (F: 5′-GACCTCGGACCTGTTGGGTAC-3′ and R: 5′-TACCCCATCTCCCCCTTCAC-3′) [10], and *GAPDH* (F: 5′-GCACCGTCAAGGCTGAGAAC-3′ and R: 5′-TGGTGAAGACGCCAGTGGA-3′) [11]. qPCR conditions were initiated with denaturation at 94 °C for 2 min, followed by up to 40 cycles consisting of 94 °C for 10 s, annealing at 54 °C, 58 °C, 63 °C (*COL6A1, COL1A1*, and *GAPDH,* respectively) for 30 s, and 72 °C for 1 min, followed by a final extension at 72 °C for 10 min. PCR products were then separated by 2% agarose gel along with a 100-bp DNA ladder (Bioron, Germany, Cat. No. 306009) (Fig. 1). Gene expression was quantified using ImageJ software for densitometry analysis. (Fig. 2).

### Statistical analysis

Data were analyzed for their normality distribution using the Kolmogorov–Smirnov test. Kruskal–Wallis and Mann–Whitney U tests were used for the data that were not normally distributed, while the normally distributed data were analyzed with one-way ANOVA tests. *p* < 0.05 was considered statistically significant.

## Results

The baseline characteristics of our patients are described in Table 1. Most patients showed midshaft and penoscrotal hypospadias (67.5%) (Table 1). qPCR showed that *COL1A1* and *COL6A1* expression was significantly downregulated in the distal (0.88 (0.38–2.53) and 0.54 (0.16–4.35), respectively) and proximal (0.76 (0.33–2.57) and 0.57 (0.18–1.38), respectively) hypospadias groups compared to controls (1.85 (0.24–4.61) and 0.93 (0.17–4.06), respectively) with *p*-values of 0.024 and 0.018, respectively (Table 2). Interestingly, *COL1A1* and *COL6A1* were also significantly downregulated in the moderate and severe chordee groups compared to the mild chordee groups, with *p*-values of 0.003 and 0.037, respectively (Table 2).

**Table 1:** Characteristics of all patients in our institution

Characteristics	Mean ± SD; N (%)	*P* value
Age (years)		
Hypospadias patients Controls	7.85 ± 3.9 5.71 ± 3.92	0.052
Hypospadias type (n = 40)		N/A
Distal		
Glandular	1 (2.5)	
Subcoronal	5 (12.5)	
Midshaft	14 (35)	
Proximal		
Penoscrotal Scrotal Perineal	13 (32.5) 6 (15) 1 (2.5)	
Penile curvature (n = 40)		0.132
Mild (< 30 degree) Moderate (30–60degree) Severe (> 60 degree)	14 (35) 12 (30) 14 (35)	
Penoscrotal transposition		
Yes No	2 (5) 38 (95)	0.147
Bifid scrotum		
Yes No	6 (15) 34 (85)	0.376

*SD* standard deviation

Significant result *p* < 0.05

**Table 2:** Comparison of COL1A1 and COL6A1 expressions based on hypospadias type and penile curvature severity

Hypospadias type	Distal Hypospadias	Proximal Hypospadias	Control	*P*-value
COL1A1	0.88 (0.38–2.53)	0.76 (0.33–2.57)	1.85 (0.24–4.61)	0.024*
COL6A1	0.54 (0.16–4.35)	0.57 (0.18–1.38)	0.93 (0.17–4.06)	0.018*
Penile curvature Severity	Mild (n = 14)	Moderate (n = 12)	Severe (n = 14)	*P*-value
COL1A1	1.56 (0.47–2.57)	0.69 (0.33–1.59)	0.73 (0.38–1.11)	0.003*
COL6A1	0.90 (0.16–4.35)	0.57 (0.23–1.04)	0.44 (0.18–0.89)	0.037*

^*^, significant result *p* < 0.05 (Kruskal Wallis test)

Moreover, there was no significant difference in COL1A1 and COL6A1 expression between proximal and distal hypospadias or severe and moderate penile curvature (Table 3). Furthermore, there was a moderate correlation between *COL1A1* and *COL6A1* expression (*r* = 0.458, *p* = 0.000).

**Table 3:** Comparison of COL1A1 and COL6A1 expressions between hypospadias groups, with moderate and severe penile curvature

Gene	Hypospadias type		*p*	95% CI		Penile Curvature		p*	95% CI	
				Lower	Upper				Lower	Upper
COL1A1	Distal	Proximal	0.957	0.963	0.972	Moderate	Severe	0.877	0.896	0.911
COL6A1	Distal	Proximal	0.570	0.570	0.596	Moderate	Severe	0.123	0.125	0.142

*Mann–Whitney test; CI, confidence interval; significant result *p* < 0.05

## Discussion

Here, we are able to show downregulated *COL1A1* and *COL6A1* expression in hypospadias patients with moderate and severe penile curvature. These results are consistent with previous research, which found that the mean number of total collagen fibers in dartos tissue in hypospadias was lower but had thicker fibers compared to normal patients [9]. They also found that the hypospadias tissue anomaly consisted of a thick and inelastic tissue, although it was not always followed by an increase in collagen [9]. Another study showed no evidence of fibrous bands or dysplastic tissue in subepithelial biopsies analyzed in 17 prepubertal boys undergoing hypospadias repair [12]. Eros et al*.* proposed no difference in collagen intensity between normal areas and under the urethral plate of patients with hypospadias [13].

Type 1 collagen is the most abundant collagen. It is the predominant component of interstitial membranes. It is also associated with fibrosis and fibrogenesis [14]. Hayashi et al. [6] revealed that during the maturation stage of scar formation, the collagen subtype I is produced excessively, developing the fibrils with large and stiff bands. This process might be associated with the less tumescence in the penis with fibrosis of the corpus cavernosum [6].

This paper also investigated the gene expression of *COL6A1* as the most studied type of collagen in the beaded filament-forming collagen group. It plays a central role in cell attachments and connections between tissues and the surrounding matrix [15]. Type 6 collagen also regulates fibrogenesis by modulating the interactions among cells. It stimulates the activation of mesenchymal cells into myofibroblasts, resulting in extracellular matrix deposition and tissue fibrosis [15]. In lung fibrosis, type 6 collagen is increasingly expressed [15]. However, this study found decreased gene expression of COL6A1 in the hypospadias group compared to the control group. This finding might be related to the downregulation of COL1A1, which was found in this paper, suggesting that type 6 collagen is bound together to the sides of type 1 collagen to form thicker collagen fibers [7]. We found a significant positive correlation between COL1A1 and COL6A1.

The etiology of chordee in hypospadias remains unclear. Resection of dartos tissue usually can straighten the penis in patients with chordee and buried penis, suggesting that the pathophysiology of these anomalies is related to dartos tissue [4]. The composition of Dartos fibromuscular tissue determines tissue elasticity and skin mobility [4, 12].

Collagen is a part of the tissue backbone. During tissue turnover, it is formed and degraded to maintain tissue health and homeostasis. Imbalance of that process leads to fibrosis. Fibrosis is known as excessive formation of connective tissue, which damages the structure and function of its tissue [16]. Chordee occurs because of fibrosis in darto tissue, which disrupts tissue elasticity and results in penis curving [17].

We also investigated the significant difference in *COL1A1* and *COL6A1* expression among groups based on penile curvature in the hypospadias group. Mostly, severe forms of hypospadias are related to a significant chordee and a urethral meatus located proximal to the midshaft of the penis [18]. Another study proposed that the severity of chordee is generally proportional to the degree of hypospadias [19]. Many factors, such as abnormal development of the urethral plate, fibrotic mesenchymal tissue at the urethral meatus, and ventral-dorsal corporal disproportion, may be interconnected, resulting in different degrees in the final severity of curvature in each patient [20]. The Dartos tissue pattern is not influenced by age. Race and genetics may affect penis development and need further investigation [4]. Finally, for clinical implications, one of the important steps before urethroplasty is the release of fibrous tissue. The urethral plate malformation might be not associated with the most penile curvature [6]. Therefore, leaving the urethral plate is preferred by most hypospadiologists [6]. In addition, removal of the tissue under the urethral plate is still debatable [6], while excision of the inelastic dartos tissue during reconstructive surgery is recommended [9].

## Conclusion

Aberrant *COL1A1* and *COL6A1* expression might affect abnormalities in darto tissue and penile curvature severity in hypospadias patients.

## Data Availability

All data generated or analyzed during this study are included in the submission. The raw data can be requested to the corresponding author.

## Abbreviation

ECM: Extracellular matrix
COL1A1: Collagen 1 subtype alpha 1
COL6A1: Collagen 6 subtype alpha 1
GAPDH: Glyceraldehyde 3-phosphate dehydrogenase
qPCR: Reverse-transcriptase polymerase chain reaction
ECM: Extracellular matrix
COL1A1: Collagen 1 subtype alpha 1
COL6A1: Collagen 6 subtype alpha 1
GAPDH: Glyceraldehyde 3-phosphate dehydrogenase
qPCR: Reverse-transcriptase polymerase chain reaction
